# Surgical treatment of type A acute aortic dissection with cerebral malperfusion: a systematic review

**DOI:** 10.1186/s13019-022-01894-8

**Published:** 2022-06-03

**Authors:** Changtian Wang, Lei Zhang, Tao Li, Zhilong Xi, Haiwei Wu, Demin Li

**Affiliations:** Department of Cardiovascular Surgery, Jinling Hospital, Nanjing University, School Medicine, 305 East Zhongshan Road, Nanjing, 210002 People’s Republic of China

**Keywords:** Type A acute aortic dissection, Cerebral malperfusion, Surgical treatment, Outcome

## Abstract

**Objectives:**

Type A acute aortic dissection (TAAAD) complicated with cerebral malperfusion (CM) is a life-threatening condition associated with high mortality, poor outcomes, and the optimal surgical management remains controversial. The aim of this review was to report the current results of surgical interventions of these patients.

**Methods:**

A systematic review was performed using PubMed and MEDLINE search for cases underwent surgical repair for TAAAD with CM. Demographics, neurological symptom, the time from onset of symptoms to operation, operation data, mortality, neurological outcome, and follow-up were reviewed.

**Results:**

A total of 363 patients with mean age of 65.7 ± 13 years underwent surgical repair for TAAAD with CM were identified in 12 retrospective studies. In-hospital mortality was 20.1%. Mean duration of follow-up was 40.1 ± 37.6 months. The involved supra-aortic branch vessels were RCCA (n = 99), LCCA (n = 25), B-CCA (n = 52), CCA (n = 131), IA (n = 19), and LSA (n = 8). Time from onset of neurological symptoms to surgery was 13.3 h. Antegrade and/or retrograde cerebral perfusion were applied. Postoperatively, improved, unchanged and worsened neurological status was occurred in 54.3%, 27.1%, and 8.5%, respectively in 199 patients.

**Conclusion:**

The outcomes of surgical treatment of TAAAD complicated with CM indicate acceptable early mortality and morbidity. It is reasonable to perform lifesaving surgery on these patients. Early central surgical repair and reperfusion of brain may improve the outcomes.

## Introduction

Type A acute aortic dissection (TAAAD) is a life-threatening condition associated with high mortality that requires emergency surgery. Malperfusion of aortic branch vessels is both common and catastrophic, affecting up to one-third of TAAAD cases, and strongly predicting poor outcomes [1]. Cerebral malperfusion (CM) secondary to the occlusion or stenosis of the supra-aortic trunks in TAAAD is an especially feared complication, significantly worsens survival and postoperative quality of life [2, 3]. The incidence of CM in TAAAD has been reported between 6% and 26% in single center studies [3–6]. The mortality associated with the surgical management of TAAAD with CM is high, and the outcomes from previous series have been mixed. To date, the optimal management of TAAAD complicated with CM remains controversial despite numerous advances in the past decades. Coma or stroke complicating TAAAD was once considered to be an absolute contraindication to surgery [7]. Urgent surgical repair in the presence of TAAAD complicated CM can prevent the early death due to aortic rupture, organ malperfusion, or complicated acute aortic valve insufficiency, but has the risk of hemorrhagic worsening of ischemic infarction during CPB after reperfusion and before CPB and full anticoagulation. Recently, reports have applied the aggressive surgical approach for patients with TAAAD complicated with CM and shown acceptable outcomes [5, 8, 9].

The aim of the present systematic review is to investigate the current status of the management strategy of TAAAD patients complicated with CM. Understanding these outcomes is helpful for the choice of optimal management of TAAAD patients complicated with CM.

## Methods

### Literature search strategy

This systematic review was performed and reported in line with the Preferred Reporting Items for Systematic Reviews and Meta-Analyses statement [10]. We searched in PubMed and MEDLINE (https://www.ncbi.nlm.nih.gov/pubmed/) with time point set to end of May 2021 using medical subject headings and text words supplemented by scanning the bibliographies of recovered articles included “type A acute aortic dissection”, “acute type A dissection”, “DeBakey Type I/II aortic dissection“”, cerebral malperfusion”, “brain malperfusion”, “stroke”, “coma”, “neurological complications”, and using the Boolean word “AND”. Prior to data extraction, all titles, abstracts and full texts were sequentially reviewed following inclusion criteria. Moreover, the reference lists of the selected articles were manually screened and reviewed to identify further relevant citations. Two co-authors (CW and LZ) reviewed and selected relevant articles for inclusion. Differences were resolved in consensus discussions. In order to avoid duplicates, all included studies were independently assessed and critically evaluated. Additional hand searching was undertaken.

### Definition

CM was defined by clinical presentation at the time of TAAAD with clear evidence of neurologic deficit on physical examination. Clinical deficits were associated with preoperative symptomatic motor or sensory deficits, including transient neurological deficit, syncope, stroke, coma, altered consciousness, and hemiplegia.

### Inclusion criteria

The predefined inclusion criteria were cases underwent surgical repair for TAAAD complicated with CM confirmed by contrast-enhanced CT or carotid duplex scan prior operation. The reports in which we could extract the exact number of cases with TAAAD complicated with CM from the total amount of reported cases were included. It was essential that included studies should document the data on operation and outcomes, including mortality, neurological outcomes and follow-up. Only the most recent report from each centre was accepted in order to avoid duplicate data. Language was limited to articles written in English.

### Exclusion criteria

The articles in which CM was confirmed during or after surgical repair were excluded. Papers in which data could not be precisely extracted were also excluded. The reports which could not be reviewed by fulltext were not included. Case reports, correspondence letters, expert opinions and reviews were not included, as well.

### Data extraction and risk of bias assessment

We collected data on study design, number of patients, age, gender, preoperative neurological symptom including syncope, stroke, hemiplegia, comatose state, Glasgow Coma Scale (GCS), and National Institutes of Health stroke scale (NIHSS), the time from onset of symptoms to operation, the operation data including cannulation, procedure (hemiarch replacement, HAR; partial arch replacement, PAR; total arch replacement, TAR), hypothermic cardiac arrest (HCA) time, temperature during HCA, cerebral perfusion (antegrade cerebral perfusion, ACP; retrograde cerebral perfusion, RCP), cross-clamp time (CCT), and cardiopulmonary bypass (CPB) time, the mortality rate, the neurological outcomes and follow-up.

The risk of bias was assessed at the study level using the Cochrane’s Collaboration Risk of Bias Tool [11]. Through six domains, this tool evaluates the risk of bias and categorizes each study as high-risk, low-risk, or unclear-risk of bias.

### Statistical analysis

Data collected were organized in an Apple Numbers spreadsheet version 6.6.2. A descriptive statistics was used to describe the demographic data with continuous variables reported as mean ± standard deviation (SD) and dichotomous variables expressed as numbers with percentage.

## Results

**Fig. 1:**
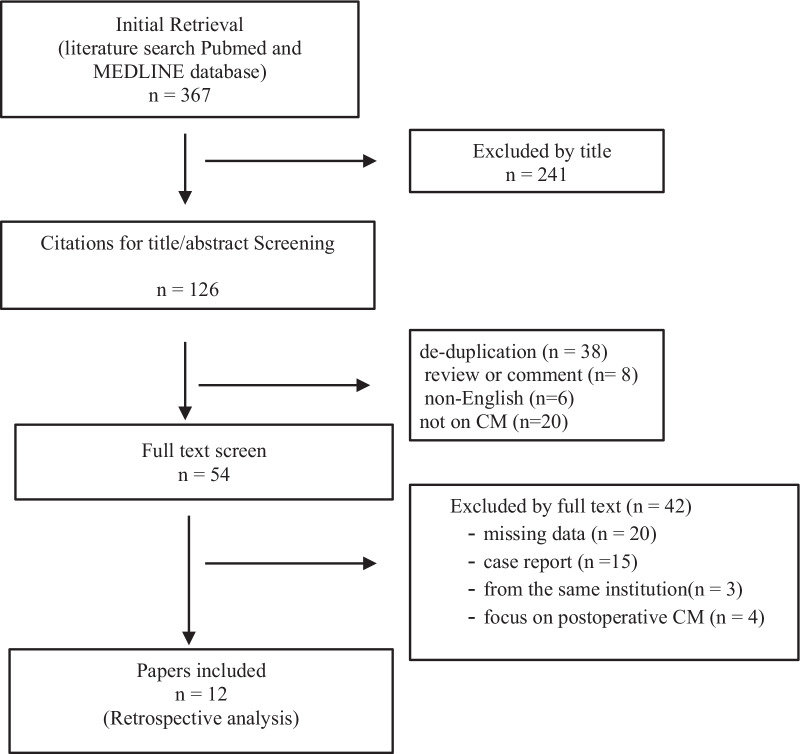
Flow diagram to illustrate identification, selection and exclusion of articles used for the review. *CM* cerebral malperfusion

The literature search yielded 367 publications in PubMed and MEDLINE databases. We screened them by title/abstract and full text. As a consequence of this analysis, 12 publications focusing on the treatment of TAAAD complicated with CM were included in the study, spanning a period of time ranging from 2005 to 2021 (Fig. 1). All included reports were retrospective studies.

### Patient characteristics

A total of 2342 patients underwent surgical repair for TAAAD. Of these, 363 (15.5%) presented with CM. 100 (27.5%) were females, and the mean age was 65.7 ± 13 years [3, 5, 8, 9, 12–19]. All patients were confirmed TAAAD with CM by clinical presentation and contrast-enhanced CT or carotid duplex scan, and received surgical repair for TAAAD. No studies reported the preoperative EuroSCORE II or the American Society of Anesthesiologists score.

### In‐hospital mortality, cause of death and follow-up

In-hospital mortality was 20.1% (n = 73), ranging from 0 to 50%. 10 studies (n = 222) documented the causes of death: severe brain damage or neurological (n = 28/222), multiorgan failure (n = 1/222), aortic rupture (n = 1/222), acute myocardial infarction (n = 1/222), and small bowel necrosis (n = 1/222). The median time from onset of neurological symptoms to surgery was 13.3 ± 31.3 h, ranging from 0.6 h to 10 days. Of these, 214 cases were within 10 h, and 149 cases were > 10 h. The mortality was 16.7% and 23.4% respectively. Mean duration of follow-up was 40.1 ± 37.6 months in 10 papers for 302 patients. Of these studies, the five-year survival was 65.3% ± 11% in 5 papers, and the 10-year survival was 60.5 ± 23.4% and 59% ± 9% respectively in two studies (Table 1).

**Table 1:** Case series summary of surgical treatment of TAAAD complicated with CM

Author	Article types	Year	N of patients	Incidence (%)	Age (years)	Sex (F)	In-hospital mortality (%)	Causes of death	Follow-up (mo)
Okita et al. [12]	RS	2021	50	13.1	68.1 ± 9.2	NA	14 (28%)	Stroke 8	5-year survival (75.2 ± 12.5%) 10-year survival (60.5 ± 23.4%)
Sugiyama et al. [13]	RS	2021	19	21	69 (39–84)	2	2 (10.5%)	Extensive cerebral infarction	NA
Gomibuchi et al. [14]	RS	2021	42	21.3	64.9 ± 11.1	22	2 (4.8%)	NA	NA
Sasaki et al. [8]	RS	2020	9	7.1	66.2 ± 12.9	8	0	0	24 (all survival)
Shimura et al. [15]	RS	2020	16	7.7	63 (32–83)	8	1 (6.3%)	Severe cerebral edema	101 ± 7
Luehr et al. [16]	RS	2016	23	6.5	66.3 (55.2–69.9)	5	3 (13.0%)	Cerebral haemorrhag 2, MOF 1	15.2 (4.8–34.1)
Di Eusanio et al. [3]	RS	2013	99	7.5	63.1 ± 13.7 62.8 ± 12.2	NA	33 (33.3%)	NA	Median 36 5-year survival: 67.1% (CVA), 57.1% (coma)
Morimoto et al. [5]	RS	2011	41	26.1	67.3 ± 10.0, 69.7 ± 11.5	16	6 (14.6%)	Large hemispheric infarction	57.6 (1.2–136.8) 5-year survival 65% ± 8% 10-year survival 59% ± 9%
Nakamura [17]	RS	2011	10	22.4	69 ± 9 (57–84)	5	0	0	18 ± 5
Tsukube et al. [18]	RS	2011	24	14.9	71.9 (44–91)	18	4 (16.7%)	Aortic rupture 1, AMI 1, massive brain edema 2	34.5 ± 25.1 3 years cumulative survival rate 71.8%
Estrera et al. [9]	RS	2006	14	19	56 (43–73)	6	1 (7%)	Small bowel necrosis	18.5 1-year survival 81.3% 5-year survival 58.0%
Tanaka et al. [19]	RS	2005	16	25.3	71.6 ± 7.7	10	7 (43.7%)	Severe brain damage	25.2 ± 21 (2–56) 4-year survival 50.1%
Total	RS	2005–2021	363 (15.5%)	15.9	65.7 ± 13 (32–91)	100	73 (20.1%)	Neurological damage (n = 28), MOF (n = 1), aortic rupture (n = 1), AMI (n = 1), small bowel necrosis (n = 1)	40.1 ± 37.6 5 years survival 65.3 ± 11%

AMI = acute myocardial infarction; CM = cerebral malperfusion; mo = month; MOF = multiorgan failure; NA = not available; TAAAD = Type A acute aortic dissection

### Preoperative neurological presentation and postoperative neurological outcomes

The documented preoperative neurological presentation was in 73.3% of patients. The most presentation was stroke or cerebrovascular accident (n = 188) and coma or altered consciousness (n = 124). Syncope presented in 54 patients, and TIA presented in 16. The rare presentation seizure was in 3 patients. The preoperative GCS was reported in 3 studies, but the definition is varied. The preoperative NIHSS score was 18.2 ± 13.3 in 3 papers. One paper provided a Japan Coma Scale [2 (n = 6), 20 (n = 2), 200 (n = 1)].

In nine studies, the postoperative neurological outcome was documented according to the recovery of neurologic status including 199 patients, completely recovered or improved in 108 (54.3%) patients, remained the same or on change in 54 (27.1%) patients, and worsened in 17 (8.5%) patients. Two reports including 66 CM patients only recorded the new-onset postoperative neurological deficits (n = 30, 45.5%), and the recovery of neurology was not documented. In one paper, the post-CVA was 11 (11.1%), and the post-coma was 9 (9.1%). The details of preoperative neurological presentation and outcomes in TAAAD complicated with CM underwent surgical treatment were summarized in Table 2.

**Table 2:** The preoperative neurological presentation and outcomes in TAAAD complicated with CM underwent surgical treatment

Author	N of patients	PreNS	Neurological state (GCS/NIHSS)	Involved SABV	Neurological outcomes
Okita et al. [12]	50	TIA 10 Coma/altered consciousness 12 Hemiplegia 28	GCS: severe (3–8) 12 moderate (9–12) 18 mild (11–13) 20	RCCA 34 LCCA 2 B-CCA 14	Improved 14 No change 16 Worsened 3
Sugiyama et al. [13]	19	Neurological deficit 8	NA	CCA	Improved 3 No change 11 Worsened 5
Gomibuchi et al. [14]	42	Persistent neurological deficit 8 Transient 5 No 29	NA	RCCA 28 LCCA 5 B-CCA 9	NewPOND 16
Sasaki et al. [8]	9	Seizure 2 Hemiplegia 4	Japan Coma Scale 2(6), 20(2), 200(1)	IA or RCCA	Improved 9 No change 0 Worsened 0
Shimura et al. [15]	16	Coma 10 Hemiplegia 6	GCS: ≤ 8 (10)	IA 16, RCCA12 LCCA 7 LSA 8	Improved 14 No change 0 Worsened 2
Luehr et al. [16]	23	Syncope 7, seizure 1, vertigo 5, photopsia 1, confusion 7	NA	B-CCA 1 LCCA 10 RCCA 12	NewPOND 14
Di Eusanio et al.[3]	99	Syncope 61, CVA 87, coma 54	NA	NA	Post-CVA 11 Post-coma 9
Morimoto et al. [5]	41	Coma 7, stupor 13, hemiplegia 19, hemianopsia 2	NIHSS:10.7 ± 7.9 (median 8.0)	CCA	Improved 26 No change 15 Worsened ?
Nakamura et al. [17]	10	Hemiplegia 9, CVA 6, motor aphasia 1	NIHSS: 5.5 ± 2.9 (4–13)	IA 3 B-CCA 7	Improved 10 No change 0 Worsened 0
Tsukube et al. [18]	24	Coma 27	GCS 6.5 (3–10) NIHSS 31.4 ± 6.6	Unilateral 12 Bilateral 10	Improved 19 No change ? Worsened 1
Estrera et al. [9]	14	CVA 16 Coma 1	NIHSS: 12.9	NA	Improved 8 No change 6 Worsened 1 (< 10 h, 80% improved > 10 h, none improved)
Tanaka et al. [19]	16	Hemiplegia 8, TIA 6, coma 5, drawsy 3, deficit of consciousness 2	GCS: 9.5 ± 5.2 (16, 5; 9–11, 4; < 6, 7)	RCCA 4 LCCA 1 B-CCA 11	improved 5 No change 6 Worsened 5
Total	363		GCS (4 papers) definition is varied. NIHSS score 18.2 ± 13.3 (4 papers)	RCCA (n = 99), LCCA (n = 25), B-CCA (n = 52), CCA (n = 131), IA (n = 19), LSA (n = 8)	Improved 108 No change 54 Worsened 17 (of 199 patients)

B-CCA = bilateral common carotid artery; CCA = common carotid artery; CM = cerebral malperfusion; CVA = cerebrovascular accident; GCS = Glasgow Coma Scale; IA = innominate artery; ICA = internal carotid artery; LCCA = left common carotid artery; LSA = left subclavian artery; NA = not available; NIHSS = National Institutes of Health stroke scale; PreNS = preoperative neurological symptom; RCCA = right common carotid artery; SABV = supra-aortic branch vessels; TAAAD = Type A acute aortic dissection

### Operative details

**Fig. 2:**
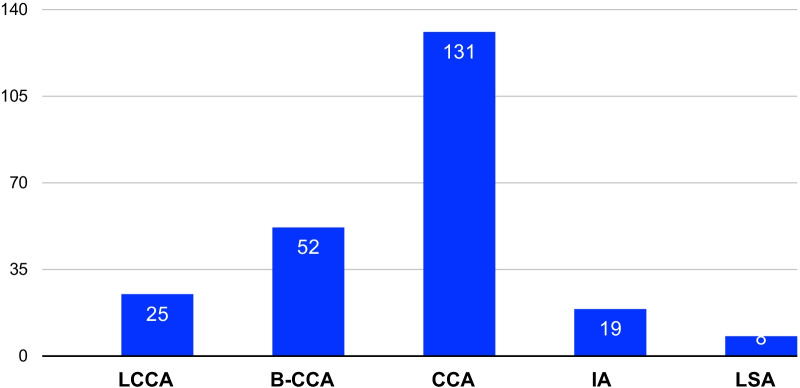
The involved supra-aortic branch vessels

The involved supra-aortic branch vessels were shown in Fig. 2. All patients underwent median sternotomy with total cardiopulmonary bypass and surgical repair of ascending aorta with or without proximal aortic root repair. The arch repair depended on the tear location. 118 (38.4%, 118/307) patients underwent hemiarch replacement (HAR), 86 (28%, 86/307) underwent total arch replacement (TAR), and 50(16.3% 50/307) underwent partial arch replacement (PAR). Two studies reported the additional procedure extra-anatomic aorto-carotid bypass for impaired CCA (n = 32). The femoral or right axillary arteries or both were the most frequent choices for arterial cannulation. The additional arterial inflow included ascending aorta (n = 22), carotid artery (n = 11), innominate artery (n = 6), cardiac apex (n = 1). Antegrade (unilateral or bilateral) and/or retrograde cerebral perfusion were (was) recorded as brain protection during surgery in ten reports including 214 patients. Of these patients, 152 (71%) underwent ACP, 67 (31.3%) underwent RCP. The mean target core temperature during hypothermic cardiac arrest was 22.7 ± 3.3 (15–29.3) °C, and the mean hypothermic cardiac arrest time was 41.9 ± 16.7 (18–77) min in 91 patients. The mean cross-clamp time and CPB time was 131 ± 45.4 and 213.9 ± 73.8 min respectively. The details of operative procedures were summarized in Table 3.

**Table 3:** Summary of details of procedures in the treatment of TAAAD complicated with CM

Author	N of patients	Time OSTOR (h)	Procedure	Cannulation (arterial inflow)	CP (ACP/RCP)	T during CA (°C)	HCA (min)	CCT (min)	CPB time (min)
Okita et al. [12]	50	6.0 ± 32.6	HAR 32 PAR 2 TAR 18	FA 32, Ax 9, AA 7, FA + Ax 3	NA	NA	NA	NA	NA
Sugiyama et al. [13]	19	5.7 (3.5–8.4) 7.1 (2.9–9.9)	HAR 3 PAR 5 TAR 11	Ax + FA	ACP 19	23.8 (21.1–24.7) 23.5 (21.8–24.5)	58 (33–77) 49 (38–65)	133 (104–199) 152 (100–236)	275 (162–378) 254 (185–355)
Gomibuchi et al. [14]	42	8.8 ± 8.2 8.0 ± 2.9	NA	Ax + FA	ACP 42	NA	NA	NA	NA
Sasaki et al. [8]	9	7.2 ± 2.4	HAR 4 PAR 0 TAR 5	IA 5, RCCA 4, Ax + FA	ACP 9	25	57 ± 15 min	118 ± 24	241 ± 44
Shimura et al. [15]	16	5.5 (2.9–9.4)	HAR 0 PAR 0 TAR 6	AA 14, AA + FA 2	RCP 16	17.5	33 (25–45)	NA	203 (128–304)
Luehr et al. [16]	23	7.0 (4.9–12.1)	HAR 14 PAR 0 TAR 9	Ax 15, FA 7, IA 1, AA 1, Cardiac apex1	ACP 23	26.0 ± 3.3	33.5 ± 14.4	111.4 ± 36.2	198.2 ± 53.6
Di Eusanio et al. [3]	99	12.3 (6.6–56.1), 13.8 (6.3–24.0)	TAR 12 PAR 42 TAR 0	NA	NA	NA	NA	NA	NA
Morimoto et al. [5]	41	21.7 ± 40.5 (median 6.5)	HAR 15 PAR 0 TAR 26	Ax, FA	ACP 19 RCP 22	23.0 ± 2.3, 22.0 ± 3.8	NA	119.2 ± 51.4, 123.0 ± 62.0	196.3 ± 76.8, 191.3 ± 67.7
Nakamura [17]	10	9.2 ± 8.7	HAR 9 PAR 0 TAR 1	NA	ACP 9 RCP 1	25 ± 1	31 ± 9	NA	144 ± 32
Tsukube et al. [18]	24	3.4 ± 1 (21) 35.5 ± 8.4(3)	HAR 19 PAR 0 TAR 5	FA 23, FA + Ax 1	ACP 27	18	NA	NA	267 ± 50
Estrera et al. [9]	14	6.1 ± 2.9 (10) 162 ± 90.6(4)	HAR ? PAR 0 TAR 0	FA, Ax	RCP 16	15—20	28 (18–46)	NA	126 (101–236)
Tanaka et al. [19]	16	7.8 ± 6.6 (3–30) [7,4 days]	HAR 10 PAR 1 TAR 5	FA 10, FA + Ax 6	RCP 12 ACP 4	20	NA	NA	NA
Total	363	13.3 ± 31.3	HAR = 118 PAR = 50 TAR = 86	AA 22, apex 1, carotid 11, IA 6	ACP = 152 RCP = 67	22.7 ± 3.3 (15–29.3)	41.9 ± 16.7 (18–77)	131 ± 45.4 (61–236)	213.9 ± 73.8 (101–378)

AA = ascending aorta; ACP = antegrade cerebral perfusion; Ax = axillary artery; CA = circulatory arrest; CPB = cardiopulmonary bypass; CCT = cross-clamp time; CM = cerebral malperfusion; CP = cerebral perfusion; ER = emergency room; FA = femoral artery; HAR = hemiarch replacement; HCA = hypothermic cardiac arrest; IA = innominate artery; LCCA Rep = left common carotid artery replacement; NA = not available; ONSTS time = the median time from onset of neurological symptoms to surgery; PAR = partial arch replacement; RCCA Rep = right common carotid artery replacement; RCP = retrograde cerebral perfusion; T = temperature; TAAAD = Type A acute aortic dissection; TAR = total arch replacement

## Discussion

In the study from the International Registry of Acute Aortic Dissection (IRAAD) reported by Sultan et al. [6], which represented the largest study to date assessing the impact of CM at the time of presentation for patients with TAAAD, 15.1% of patients presented with CM. Our study also reveals that the incidence of CM in TAAAD is 15.9%, ranged from 6.5% to 26.1%. Despite the surgical techniques, the anesthesiology and perioperative management have greatly improved over the last years, and the mortality rate has improved significantly in the surgical treatment for TAAAD patients with CM, the mortality remains high. A report by Fann JI, et al. in 1989 demonstrated that the surgical results of such patients showed a high mortality rate of 57% [20]. In 2007, the report from University of Pennsylvania by Geirsson et al. [21] showed that the mortality rate was 50% in surgical repair for TAAAD with CM patients. A report from Japan by Okita Y, et al. in 2021 showed that the mortality rate was 28% [12]. From our investigation, the mean in-hospital mortality is 20.1%. However, the outcomes are variable. In a few studies, the mortality was 0 and the long time survival was acceptable. Although the numbers of cases in these studies were not so enough, the results were encouraging in this critically ill cohort of patients. TAAAD patients with CM should not preclude surgical candidacy.

In the case of acute ischemic cerebrovascular pathology, the interval from onset of neurological symptoms to return of cerebral blood flow is a key factor in determining the severity and recovery of cerebral injury. The 2015 guidelines of the Healthcare Professionals from the American Heart Association/American Stroke Association reported the efficacy of endovascular treatment within 8 h of symptom onset for patients with acute ischemic stroke [22]. Data from our review shows that the mean time from presentation of neurological symptoms to surgical intervention is 13.3 h. Multiple studies shown patients who initially underwent early surgical repair or reperfusion of brain had good outcomes [5, 8, 9, 18, 23, 24]. The mortality in the group which surgery underwent within 10 h was significantly lower than that of patients over 10 h. Tsukube et al. [18] performed immediate surgery within 5 h for TAAAD patients with coma, which resulted in full recovery of consciousness in 86% and hospital mortality of 14%. Estrera et al. [9] reported the operative results of 16 patients with TAAAD complicated by preoperative stroke. The median time from onset of stroke to surgery was 9 h, and 80% of patients who underwent surgical repair within 10 h had improvement in neurologic status, whereas none improved if operated on beyond 10 h. Sasaki et al. [8] reported that hemiplegia and hemiparesis improved significantly after immediate aortic repair in which the time from onset of symptoms to operating room was 7.2 ± 2.4 h, with hospital mortality of 0% and overall survival at 24 months after operation of 100%. Morimoto et al. [5] also reported that 9.1 h was an optimal cutoff value for predicting lack of neurologic improvement, if surgery was performed within 9.1 h, 88% improved neurologically, with dramatically improved 5-year survival (84% vs. 33%). Those present researches indicate that early surgical repair within 10 h may improve the outcomes. However, multicenter controlled clinical trials with large samples are needed.

Cerebral malperfusion time plays a role in determining outcomes in TAAAD patients, and expeditious revascularization is crucial in the management strategy of TAAAD with CM. Early reperfusion and extra-anatomic revascularization may reduce the risk of neurological complications. A number of institutions have adopted strategies to minimize cerebral malperfusion time and reperfuse the cerebral blood flow sooner, including extra-anatomic revascularization [8, 14, 16], direct carotid artery cannulation [12, 13, 25, 26], or percutaneous endovascular carotid artery stenting [25]. Immediate central aortic repair and primary entry tear resection is the most widely practiced early reperfusion strategy, wherein the goals are to expand the true lumen by redirecting flow into it and to decompress the false lumen by resecting the entry tear, and has been shown to improve outcomes in patients with malperfusion [9, 23, 24]. Arterial cannulation sites are determined according to a patient’s status, preoperative involved supra-aortic branch vessels and the preference of the surgeon. The right axillary artery is the most frequent choice because it will allow for uninterrupted ACP during arch reconstruction. RCP, another brain protection strategy, is also performed in many centers, which can flush out air and atheromatous debris within the arch vessels [5, 6, 9, 12, 15, 19, 25, 27]. The extent of aortic replacement is determined on the position of the primary entry, and ascending aorta combined with hemi-arch replacement with or without root replacement or repair is performed in most patients. It is necessary to investigate the correlation between outcomes and operation strategies, such as the extent of aortic replacement and selective cerebral perfusion, in TAAAD with CM patients.

Coma is the common presentation following TAAAD complicated with CM, and its definition is varied among studies. There has controversial with regards to the surgical management required cardiopulmonary bypass, full anticoagulation with hypothermic circulatory arrest for a patient in coma. The threat of use of high-dose heparin, hemorrhagic conversion of the ischemic infarction, cerebral reperfusion leading to worsening of neurologic outcome exists. For this reason, Fukuda et al. advocated intentional delay of surgical repair [28]. Fukuhara S and colleagues [25] found that all patients developed severe cerebral edema and herniation syndrome died regardless of the surgical management. Caution is necessary because the differentiation of coma is vitally important, and is difficult also. Cranial computed tomographic scanning often does not identify early acute ischemic infarction, however, is the best means to rule out acute bleeding [29]. Patients with the evidence of intracranial hemorrhage have been an absolute contraindication to immediate surgical repair.

Several limitations to our study exist. This is a retrospective systematic review of published reports on surgical treatment of TAAAD complicated with CM. The inherent limitations of a retrospective study and review should be acknowledged. There could be a risk of publication bias because several data are missing during the investigation. There is a degree of heterogeneity in the pathology, the operative and cerebral protection strategy for TAAAD patients with CM among different institutions. In addition, there is a lack of standardization in evaluation and document of the neurological presentations and outcomes. Lastly, the prognosis of TAAAD is determined on many factors, and malperfusion of other organ systems is common in TAAAD patients with CM and potential significant bias by this fact may exist. Further investigation and clinical research using standardized methodology is highly warranted to validate our results.

## Conclusion

The outcomes of surgical treatment of TAAAD with CM have been improved, and indicate acceptable early mortality and morbidity in this critically ill cohort of patients. It is reasonable to perform lifesaving surgery on patients who present with TAAAD complicated by CM. Early central surgical repair and reperfusion of brain may improve the outcomes. Indeed, clinical trials with large samples are needed.

## Data Availability

Data are available in PubMed (see references).
